# Plants use identical inhibitors to protect their cell wall pectin against microbes and insects

**DOI:** 10.1002/ece3.6180

**Published:** 2020-03-12

**Authors:** Roy Kirsch, Esma Vurmaz, Carolin Schaefer, Franziska Eberl, Theresa Sporer, Wiebke Haeger, Yannick Pauchet

**Affiliations:** ^1^ Department of Entomology Max Planck Institute for Chemical Ecology Jena Germany; ^2^ Department of Biochemistry Max Planck Institute for Chemical Ecology Jena Germany; ^3^ Research Group Sequestration and Detoxification in Insects Max Planck Institute for Chemical Ecology Jena Germany

**Keywords:** arabidopsis, leaf beetle, pectin, plant–insect interactions, polygalacturonase, polygalacturonase‐inhibiting protein

## Abstract

As fundamentally different as phytopathogenic microbes and herbivorous insects are, they enjoy plant‐based diets. Hence, they encounter similar challenges to acquire nutrients. Both microbes and beetles possess polygalacturonases (PGs) that hydrolyze the plant cell wall polysaccharide pectin. Countering these threats, plant proteins inhibit PGs of microbes, thereby lowering their infection rate. Whether PG‐inhibiting proteins (PGIPs) play a role in defense against herbivorous beetles is unknown. To investigate the significance of PGIPs in insect–plant interactions, feeding assays with the leaf beetle *Phaedon cochleariae* on *Arabidopsis thaliana pgip* mutants were performed. Fitness was increased when larvae were fed on mutant plants compared to wild‐type plants. Moreover, PG activity was higher, although *PG* genes were downregulated in larvae fed on *PGIP*‐deficient plants, strongly suggesting that PGIPs impair PG activity. As low PG activity resulted in delayed larval growth, our data provide the first in vivo correlative evidence that PGIPs act as defense against insects.

## Graphical abstract

Whether polygalacturonase‐inhibiting proteins (PGIPs) play a role in defense against herbivorous beetles is unknown. Feeding assays with the leaf beetle *Phaedon cochleariae* on *Arabidopsis thaliana* *pgip* mutants were performed. Fitness was increased when larvae were fed on mutant plants compared to wild‐type plants. Moreover, PG activity was higher, although PG genes were downregulated in larvae fed on PGIP‐deficient plants, strongly suggesting that PGIPs impair PG activity.

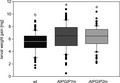

## 1 INTRODUCTION

Plants are primary producers in food webs; as such, they attract a variety of heterotrophs, for example, herbivorous insects and phytopathogenic microbes. Both of these rely on plants as their sole source of nutrients and can cause devastating effects. To counteract these effects, plants have evolved an intricate defense system comprising various chemical and physical barriers. Those include, among others, secondary metabolites and specialized morphological structures such as plant cell walls (PCWs) (Bennett & Wallsgrove, 1994; Hanley, Lamont, Fairbanks, & Rafferty, 2007). Additionally, plants produce defensive proteins: By reducing their palatability, these proteins disrupt attackers’ nutrition (Fürstenberg‐Hägg, Zagrobelny, & Bak, 2013; War et al., 2012). Inducible in response to stress, these proteins also interfere with digestive enzymes and the subsequent absorption of nutrients (Bowles, 1990; Duffey & Stout, 1996).

Well‐characterized examples of plant defense proteins are inhibitors of insect amylases and proteases, which have been extensively studied and shown to impair starch and protein digestion in the insect's gut (Jongsma & Bolter, 1997; Kaur, Kaur, & Gupta, 2014). Targeting these digestive enzymes with specific inhibitors negatively affects growth, development, survival, and fecundity, emphasizing their relevance and impact on the insect's life (Franco, Rigden, Melo, & Grossi‐de‐Sa, 2002; Jongsma & Beekwilder, 2011; Zhu‐Salzman & Zeng, 2015).

Whereas amylases and proteases are widespread among insects and their significance has been evident for decades, recent advances in sequencing technologies and bioinformatics analyses of genomes and transcriptomes have revealed the presence of several endogenous genes encoding plant‐cell‐wall‐degrading enzymes (PCWDEs) in insects. These genes include various families of glycoside hydrolases, esterases, and lyases, and have been detected in several herbivorous lineages (Calderon‐Cortes, Quesada, Watanabe, Cano‐Camacho, & Oyama, 2012; Hearn et al., 2019; McKenna et al., 2019; Wybouw, Pauchet, Heckel, & Leeuwen, 2016). PCWDEs break down PCW polysaccharides such as cellulose, hemicelluloses, and pectins. The most expanded PCWDE gene family in insects encodes polygalacturonases (PGs) that belong to glycoside hydrolase family 28 (GH28) and degrade the galacturonic acid‐rich backbone of pectin (Celorio‐Mancera et al., 2008; Kirsch et al., 2014; Shelomi et al., 2016). Pectin is highly abundant in every primary PCW and plays the role of a polysaccharide matrix, embedding cellulose and hemicellulose fibers of the PCW (Caffall & Mohnen, 2009; Voragen, Coenen, Verhoef, & Schols, 2009).

Herbivorous beetles of the Phytophaga lineage include the species‐rich weevils, long‐horned beetles, and leaf beetles (Marvaldi, Duckett, Kjer, & Gillespie, 2009). Enzymatic characterization and phylogenetic analyses of the Phytophaga GH28 family revealed massive gene duplication and a remarkable degree of subfunctionalization following the horizontal acquisition of a microbial GH28 gene (Keeling et al., 2013; Kirsch et al., 2014; Kirsch, Heckel, & Pauchet, 2016; McKenna et al., 2016). This is in contrast to what is known from phytopathogens. Upon infection, microbes secrete their PGs into the extracellular space, leading to a loosening of the PCW and the maceration of plant tissue, and, most important, to the release of nutrients (Lagaert, Belien, & Volckaert, 2009; Martens‐Uzunova & Schaap, 2008; Richard & Hilditch, 2009).

To protect their PCW polysaccharides from degradation, plants have evolved numerous inhibitors of microbial PCWDEs (Caffall & Mohnen, 2009; Lagaert et al., 2009). Among those, PG‐inhibiting proteins (PGIPs) counteract pectin hydrolysis by microbial PGs (De Lorenzo, D'Ovidio, & Cervone, 2001; D'Ovidio et al., 2004; Federici, Matte, Fernandez‐Recio, Tsernoglou, & Cervone, 2006). PGIPs are widely distributed in plants, and the number of genes encoding them in dicots ranges from two in *Arabidopsis thaliana* to 16 in *Brassica napus* (Ferrari, Vairo, Ausubel, Cervone, & Lorenzo, 2003; Hegedus et al., 2008). The PG‐PGIP interaction is an efficient mode of defense for plants because phytopathogens become less infective when their PGs are inhibited, and plants in turn show an increased level of resistance when overexpressing certain PGIPs (Hwang et al., 2010; Kalunke et al., 2015; Powell et al., 2000).

Since the seminal work by Agrawal in 1999 (Agrawal, 1999), evidence has increasingly shown that phytopathogens and herbivorous insects induce overlapping defense responses. This insight, along with our broadening understanding of the evolutionary origin, enzymatic function, and distribution of insect PGs, gives rise to the question of whether PGIPs play a dual role defending plants against microbes as well as herbivorous insects. There is some evidence that PGIPs, just by being used in excess or semipure forms, can inhibit insect PG activity (Doostdar, McCollum, & Mayer, 1997; D'Ovidio et al., 2004; Frati, Galletti, Lorenzo, Salerno, & Conti, 2006). However, the impact of PGIPs on insect–plant crosstalk in vivo has yet to be investigated.

We addressed this issue by dissecting the response of the leaf beetle *Phaedon cochleariae* to the PGIP composition of the food plant *A. thaliana*. *Phaedon cochleariae* is an oligophagous leaf beetle that feeds on various plants of the family Brassicaceae (Kühnle & Müller, 2009). The genome of this beetle encodes nine GH28 proteins, four of which possess PG activity; the remaining proteins are hypothesized to be PG pseudoenzymes (Kirsch et al., 2014). Together with wild‐type (wt) plants with two inherent functional *PGIP* genes, two *A. thaliana* mutant lines were used, one of whose *pgip*s had been knocked out. The *PGIP1* knockout line was previously shown to be hypersusceptible to nematode parasites, and moreover, the silencing of *PGIP1* enhanced fungal infection compared to wild‐type plants (Federici et al., 2006; Shah et al., 2017). Here, feeding assays followed by growth rate recordings, enzymatic assays, and real‐time quantitative PCR (RT‐qPCR) revealed that PGIP composition was correlated with larval fitness, pectin digestion, and GH28 gene expression. More precisely, PG activity was higher in larvae feeding on the *PGIP* knockout plants than in those feeding on the wt plants. The absence of one PGIP had a beneficial effect on larval growth, indicating a generally negative effect of the PGIPs on the beetle's fitness. Our work provides the first in vivo correlative evidence that PGIPs are of biological relevance for herbivorous beetles and that they contribute to the defense response of plants to both microbes and insects.

## 2 MATERIALS AND METHODS

### 2.1 Insects and plants

*Phaedon cochleariae* used for these experiments descended from a laboratory breeding stock reared on Chinese cabbage *(Brassica rapa* ssp. *pekinensis)* for more than 20 generations (15°C, 16‐hr/8‐hr light/dark period, 60% humidity). Seeds of *A. thaliana pgip* T‐DNA insertion mutants were ordered from the Nottingham Arabidopsis Stock Center (*AtPGIP1*m: GK‐092G09 [SET]; *AtPGIP2*m: GK‐717A02 [SET]), both in the Col‐0 genetic background. The GK (GABI‐Kat (Kleinboelting, Huep, Kloetgen, Viehoever, & Weisshaar, 2012)) sets were screened for homozygosity by PCR using genotyping primers (Table S1). Additionally, genomic DNA from each line and complementary DNA from RNA extractions were subjected to PCR using primers amplifying full‐length *pgip* genes and full‐length open reading frames, respectively, to verify the knockout (Figure S1). *Arabidopsis* wt plants and *PGIP* mutants were grown under standard conditions (21°C, 12‐hr light/12‐hr dark period, 50%– 60% humidity) prior to feeding assays.

### 2.2 Feeding assays and recordings of weight gain

Plants were grown in individual pots encased within transparent tubes that were covered with white gauze right after beetle larvae were added. *Phaedon cochleariae* neonates were put onto five‐week‐old *A*. *thaliana* wt plants or *PGIP* knockout mutants (three larvae per plant, 40 plants per line). Larvae were reared until the third instar (21°C, 16 hr/8 hr light/dark period, 60% humidity). Weight gain was calculated as the difference between the weight of a third‐instar larva and the average weight of a neonate. The average weight of neonates was used, since they were too small to be weighed individually, and was calculated to be 0.14 mg (average of *N* = 100 measurements, standard error of the mean (*SEM*) = ±0.017 mg).

All samples for protein and RNA extractions, as well as for metabolite analyses, were taken after a 20‐hr feeding period of third‐instar larvae under the same conditions as described above.

### 2.3 Protein extraction and PG activity analysis

Larval guts were separated from remaining tissues of prechilled larvae and subsequently opened on one side. The complete gut content from five individuals for each biological replicate was pooled to have enough starting material and centrifuged, and the supernatant was further used for enzymatic assays. The gut tissues were used for RNA isolation. Protein concentrations of gut content samples were determined by Bradford assay (Bradford, 1976) using the protein assay dye reagent (Bio‐Rad). Quantitative assays measuring the release of reducing sugars after the hydrolysis of polygalacturonic acid were set up in three biological replicates per plant line and analyzed as described previously (Kirsch et al., 2016; Miller, 1959). PG activity was expressed as nmol galacturonic acid equivalents released/min/µg of gut content protein.

### 2.4 RNA extractions from *P. cochleariae* and GH28 expression analysis

To compare *GH28* gene expression in larvae that varies depending on their diet, RT‐qPCR was performed on a CFX Connect™ Real‐Time PCR Detection System (Bio‐Rad). RNA extractions from gut tissues were done with the innuPrep DNA/RNA Mini Kit (Analytik Jena AG) following the manufacturer's instructions, and RNA integrity was checked on an agarose gel. From each pool, 500 ng of total RNA was reverse‐transcribed with a 3:1 mix of random and oligo‐dT20 primers following the manufacturer's instructions of the Verso 2‐Step qRT‐PCR Kit (Thermo Fisher Scientific). The PCR program was as follows: 95°C for 15 min, then 40 cycles at 95°C for 15 s, 58°C for 30 s, and 72°C for 30 s, and afterward a melt cycle from 55 to 95°C in 0.5‐s increments. Primers were designed using Primer3 (version 4.1.0) (Untergasser et al., 2012) and are listed in Table S1. Specific amplification of each transcript was verified by dissociation curve analysis. A standard curve for each primer pair was determined in the CFX Manager (version 3.1) based on Cq values (quantitation cycle) of RT‐qPCR running with a dilution series of cDNA pools. The efficiency and amplification factors of each RT‐qPCR primer pair were based on the slope of the standard curve and calculated using the qPCR Efficiency Calculator online tool (Thermo Fisher Scientific). *Elongation factor 1α* (*EF1α*; HE962191) was used as reference gene, and quantities of the genes of interest (*GOI*) were expressed as RNA molecules of *GOI*/1000 RNA molecules of *EF1α*. The Cq values were determined from two technical replicates of each of the three biological replicates per treatment.

### 2.5 RNA extractions from *A. thaliana* and PGIP expression analysis

To compare the expression of the *PGIP* genes between the three plant lines with respect to the treatments (feeding vs. control), RT‐qPCR was performed. RNA was extracted from leaves that were damaged by feeding *P*. *cochleariae* and from the undamaged leaves of control plants. We followed the protocol for extraction and RT‐qPCR as described above with some modifications. RNA was enriched for messenger RNA (mRNA) for reverse transcription to remove traces of genomic DNA from RNA extractions by using the mRNA Isolation Kit (Roche Diagnostics GmbH) according to the manufacturer's instructions. 100 mg of leaf material was used for mRNA enrichment. The RT‐qPCR program was as follows: 95°C for 15 min, then 40 cycles at 95°C for 15 s, 56°C for 30 s, and 72°C for 30 s, and afterward a melt cycle from 55 to 95°C in 0.5‐s increments. *Ubiquitin‐conjugating enzyme 21* (*UBC21*; NM_001036862) was used as a reference gene, and quantities of the genes of interest were expressed as RNA molecules of *GOI*/1000 RNA molecules of *UBC21*. The Cq values were determined from two technical replicates of each of the three biological replicates.

### 2.6 Glucosinolate, amino acid, and sugar analysis

The lyophilized shoots of plants (*n* = 8 per *A. thaliana* line) were ground in liquid nitrogen, and 20 mg was extracted with 1 ml of 80% methanol containing 50 µM 4‐hydroxybenzyl glucosinolate as internal standard for glucosinolate quantification by shaking for 2 min at 20 Hz in a TissueLyser II (Qiagen). Samples were heated (2 min at 90°C) while being shaken at 450 rpm and centrifuged (10 min at 1,600 *g*). An aliquot of 50 µl of the supernatant was used for amino acid and sugar analysis (see below). The remaining supernatant was subjected to glucosinolate extraction as described previously (Beran et al., 2014). Glucosinolate concentration was calculated relative to the peak area of the internal standard and expressed as µmol/g plant fresh weight.

For amino acid quantification, the supernatant was diluted 1:10 with water containing 10 µg/ml of a mix of ^15^N/^13^C‐labeled amino acids (Isotec) and subsequently subjected to LC‐MS/MS analysis on a C18 column (XDB‐C18, 50 x 4.6 mm x 1.8 µm; Agilent) as described previously (Crocoll, Mirza, Reichelt, Gershenzon, & Halkier, 2016). Amino acids were quantified relative to the peak area of their respective labeled standard, except for tryptophan, which was quantified relative to a phenylalanine standard using a response factor of 0.42. Soluble sugars were analyzed from the 1:10 diluted extract by LC‐MS/MS on a hydrophilic interaction liquid chromatography (HILIC) column (apHera NH_2_ Polymer; 15 × 4.6 mm, 5 μm; Supelco) as described previously (Madsen, Kunert, Reichelt, Gershenzon, & Halkier, 2015). All sugars were quantified using an external standard curve with authentic standards of glucose, fructose, sucrose, stachyose (all from Sigma‐Aldrich), and raffinose (Fluka).

### 2.7 Statistical analysis

Data were analyzed by one‐way ANOVA followed by Tukey's HSD test. If data were not normally distributed, Kruskal–Wallis test was used, followed by Dunn's post hoc test. All statistical analyses were performed using SigmaPlot 11.0 (Systat Software Inc.).

## 3 RESULTS

### 3.1 Larval performance correlates with the PGIP composition of the plant

**Figure 1:**
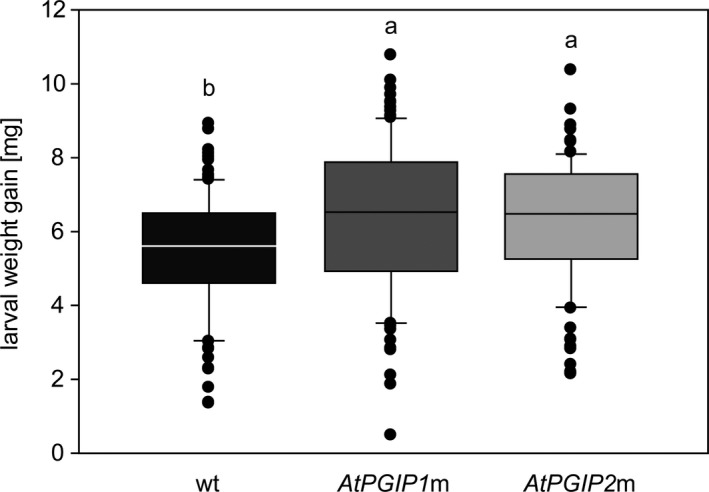
Weight gain of *P*. *cochleariae* larvae fed on *A. thaliana* wt plants and *AtPGIP* knockout mutants. Neonate larvae were fed on *A. thaliana* wt plants (black), and *AtPGIP1*m (dark gray) and *AtPGIP2*m (light gray) plants. Weight gain is displayed as the difference between the average neonate weight and their weight after 9 days (*n*_(wt) _= 101, *n*_(_ *_AtPGIP1_*_m)_ = 96, *n*_(_ *_AtPGIP2_*_m)_ = 99 larvae). Letters above each bar indicate significant differences between groups based on a Kruskal–Wallis test and Dunn's post hoc test (*p* < .05). Error bars indicate the *SEM*

The genome of *A. thaliana* encodes two PGIPs (AtPGIP1 and AtPGIP2) that are known to reduce the infection symptoms and growth of phytopathogens (e.g., *Botrytis cinerea* (Ferrari et al., 2003) and *Fusarium gramineum* (Ferrari et al., 2012)). To investigate whether PGIPs also affect the fitness of beetles known to harbor PGs, we performed feeding assays of the leaf beetle *P*. *cochleariae* on three *A*. *thaliana* lines that differ in their PGIP composition. Larval weight gain from neonate to the late third instar was determined and compared in larvae that fed on wt plants or on one of the two *A*. *thaliana PGIP* mutant lines (*AtPGIP1*m or *AtPGIP2*m). The increase in larval weight differed significantly between plant lines (Figure 1). Whereas larvae that fed on either of the knockout mutants performed equally well, the weight gain of larvae that fed on the *A*. *thaliana* wt was lower (*p *= <.001). This difference indicates that both AtPGIP1 and AtPGIP2 negatively affect larval performance as knocking out either of the proteins positively influences larval weight gain.

### 3.2 PG activity in the larval gut correlates with the PGIP composition of the plant

**Figure 2:**
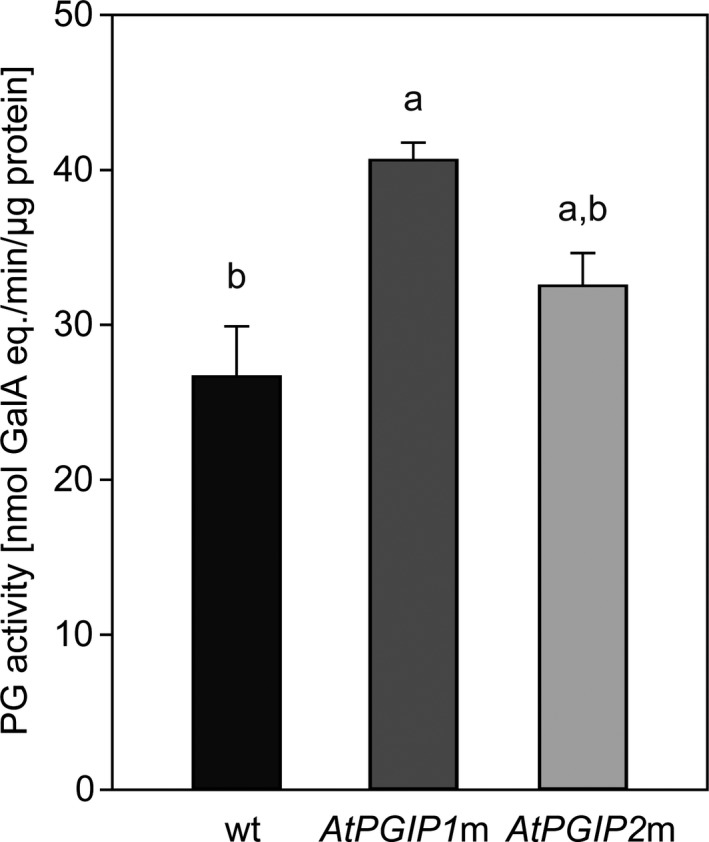
Quantification of PG activity in *P*. *cochleariae* gut content. Third‐instar larvae that fed for 20 hr on *A. thaliana* wt plants (black), and *AtPGIP1*m (dark gray) and *AtPGIP2*m (light gray) plants were dissected, and their gut PG activity was quantified (*n* = 3). Activity is expressed in nmol of galacturonic acid equivalents released per min and µg of gut content protein. Letters above each bar indicate significant differences between groups based on one‐way ANOVA followed by Tukey's HSD post hoc test (*p* < .05). Error bars indicate the *SEM*

To test whether reduced performance in larvae fed on wt plants correlates with inhibited pectin degradation in their gut, the breakdown of pectin was assayed; the assay was performed by quantifying the hydrolysis of polygalacturonic acid in vitro. The more the galacturonic acid is released from the hydrolysis of the pectin backbone, the higher the PG activity is, and thus, the more efficient the pectin digestion in larval guts should be. Indeed, PG activity was lowest in larvae that fed on *A*. *thaliana* wt plants (Figure 2). The release of galacturonic acid was significantly reduced in these larvae compared with the larvae that fed on *AtPGIP1*m (*p *= <.001). The same trend could be observed for the larvae fed on *AtPGIP2*m. Altogether, PG activity in the larval gut correlates with the PGIP composition of the food plant, and the breakdown of pectin was slower in the gut of *P*. *cochleariae* larvae which fed on *A*. *thaliana* plants expressing both PGIPs than in the gut of larvae fed on plants expressing a single PGIP.

### 3.3 The expression of both AtPGIPs is induced upon feeding

**Figure 3:**
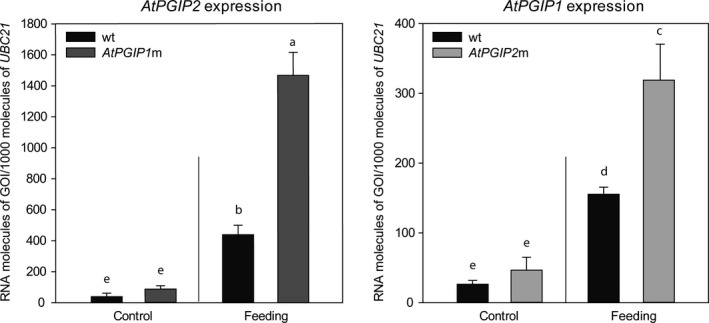
Regulation of *A. thaliana PGIP*s in response to *P. cochleariae* feeding. The expression levels of *AtPGIP2* (left) and *AtPGIP1* (right) were quantified by RT‐qPCR in wt (black), *AtPGIP1*m (dark gray), and *AtPGIP2*m (light gray) plants, respectively. Gene expression levels were compared between undamaged (control) and beetle‐damaged (feeding) plants (*n* = 3). Transcript abundances are expressed as RNA molecules of gene of interest (GOI) per 1,000 RNA molecules of the reference gene *ubiquitin‐conjugating enzyme 21* (*UBC21*). Letters above each bar indicate significant differences between groups based on one‐way ANOVA followed by Tukey's HSD post hoc test (*p* < .05). Error bars indicate the *SEM*

Both *AtPGIPs* are upregulated in response to various biotic stresses including caterpillar feeding (Appel et al., 2014). To investigate whether feeding by *P*. *cochleariae* larvae also triggers the induction of *AtPGIP* expression, we analyzed the gene expression of *AtPGIP1* and *AtPGIP2* in wild‐type and mutant plants being fed upon or not by *Phaedon* larvae. First, in response to beetle feeding, the expression of both *AtPGIPs* is significantly upregulated (*p_PGIP1 _*= <.001, *p_PGIP2_* = .004) compared with undamaged control plants in the wt line (Figure 3). The level of *AtPGIP2* transcripts was approximately three times higher than the level of *AtPGIP1* mRNA under induced conditions (*p* = .011), whereas the expression levels of both genes in control plants did not differ (steady‐state). These data show that larval feeding elicited a higher level of *AtPGIP2* than *AtPGIP1*. Second, *AtPGIP1* and *AtPGIP2* showed the same patterns of gene expression in the respective knockout mutant as in the wt plants (Figure 3). The level of expression of both genes remained similar under steady‐state conditions; their expression was induced in response to feeding (*p_PGIP1_* = .008, *p_PGIP2_* = < .001); when the mutant lines were compared, the expression of *AtPGIP2* was more highly upregulated than the expression of *AtPGIP1* (*p* = .002). However, compared with the expression of the *PGIPs* in the wt plants, the expression of the *PGIPs* in the mutant lines fed on by *Phaedon* larvae was stronger (*p_PGIP1_* = .036, *p_PGIP2_* = .003) even though steady‐state expression levels among the three plant lines did not differ significantly (Figure 3).

### 3.4 Differential gene expression of GH28s correlates with PGIP composition

**Figure 4:**
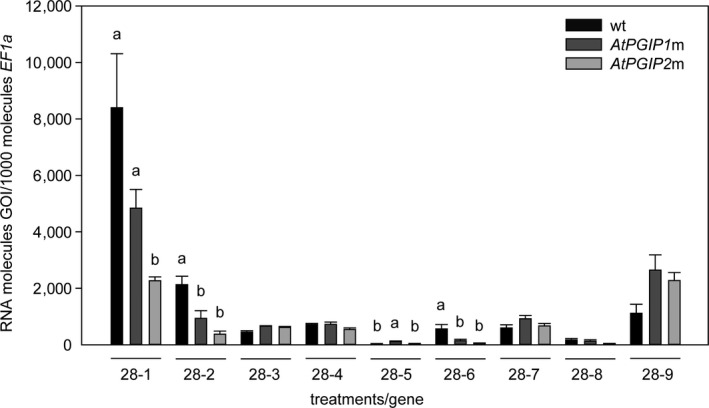
Expression pattern of *P*. *cochleariae GH28*s. Gene expression levels were quantified by RT‐qPCR and compared between larvae that fed on wt plants (black), and *AtPGIP1*m (dark gray) and *AtPGIP2*m (light gray) plants, respectively (*n* = 3). Transcript abundances are expressed as RNA molecules of gene of interest (GOI) per 1,000 RNA molecules of the reference gene *elongation factor 1‐alpha* (*EF‐1α*). Letters above each bar indicate significant differences between treatments (individually for each gene) based on one‐way ANOVA followed by Tukey's HSD post hoc test (*p* < .05). Error bars indicate the *SEM*

A hallmark of insect–plant interactions is the evolutionary arms race fueled by alternating adaptations. To test whether *P*. *cochleariae* larvae adapt to the ingestion of different PGIP compositions by altering the expression level of their *PG* genes, we compared the abundance of *PG* transcripts between larvae fed on the three *A. thaliana* lines. Among the nine *P. cochleariae* GH28s, there are both active PGs (GH28‐1, GH28‐5, GH28‐9) and PG pseudoenzymes that may have lost their activity due to substitutions of critical amino acids (Kirsch et al., 2014). As each might be involved in the interaction with PGIPs, we included them all in our analysis. Differential gene expression was observed for four out of the nine *GH28s* between larvae fed on the three plant lines (Figure 4). The expression of the gene encoding the active GH28‐5 is upregulated in larvae feeding on the *AtPGIP1*m compared with both wt (*p_wt_* = .029) and *AtPGIP2*m (*p_PGIP2_*_m_ = .042) plants. No significant differences were observed between the expressions of this gene in larvae feeding on either the wt plants or the *AtPGIP2*m plants. However, the expression of *GH28‐1*, *GH28‐2,* and *GH28‐6* was the highest in larvae fed on wt plants (*GH28‐1*: *p_PGIP2_*_m_ = .007; GH28‐2: *p_PGIP1_*_m_ = .032, *p_PGIP2_*_m_ = .006; GH28‐6: *p_PGIP1_*_m_ = .049, *p_PGIP2_*_m_ = .023). As soon as one of the PGIPs is absent from the diet, the expression of these *GH28s* is downregulated. Remarkably, the *GH28* genes that show differential expression encode both active PGs (GH28‐1) and PG pseudoenzymes of as‐yet unknown function (GH28‐2 and GH28‐6). This finding indicates that these inactive GH28s play a role in the adaptation of *P. cochleariae* to the plant PGIP defense system.

### 3.5 Plant metabolite analyses do not explain fitness differences

Plants are not equally nutritious; their protein and carbohydrate content varies as do the chemical composition and amount of defensive secondary metabolites (Bennett & Wallsgrove, 1994; Schoonhoven, Loon, & Dicke, 2005). To exclude the possibility that the differences among the phenotypes we observed for *P. cochleariae* larvae in the experiments described above were caused by differences of the nutritional value of the *A*. *thaliana* lines, we analyzed primary as well as secondary plant metabolites. Free amino acids, sugars, and glucosinolates, the last of which are known to affect insect–plant interactions (Hopkins, van Dam, & van Loon, 2009), were quantified and compared between lines after larval feeding. We found no significant differences in any of the compounds quantified and similar nutritional values in the plant lines used as food sources (Figures S2 and S3).

## 4 DISCUSSION

The GH28 polygalacturonase gene family is considerably expanded in the Phytophaga lineage, the beetles representing the largest radiation of herbivorous insects (Strong, Lawton, & Southwood, 1984). Remarkably, PGs have belonged to the digestive repertoire of those beetles since early in Phytophaga evolution, as their most recent common ancestor acquired a *PG* gene from a microbial donor by horizontal gene transfer (Kirsch et al., 2014). Consequently, beetle PGs and plant PGIPs may have started to interact ~200 MYA, when the Phytophaga lineage arose and land plants had already substantially diversified (Fiz‐Palacios, Schneider, Heinrichs, & Savolainen, 2011; McKenna & Farrell, 2009).

PGs are secreted into the gut lumen (Kirsch et al., 2012), and so, plant PGIPs can interact with them only while passing with the food bolus through the beetle's digestive tract. In a previous study, we investigated the gut content proteome of *P. cochleariae* after fractionation by anion exchange chromatography; at that time, we identified several host plant PGIPs which were eluted in the same fractions as beetle PGs (Kirsch et al., 2012). This finding not only verified that PGIPs survive the harsh environment of the beetle's gut but also indicated that PGIPs could interact with beetle PGs and were eluted because of that. Moreover, Doostdar et al. (1997) showed that a PG purified from the sugarcane weevil *Diaprepes abbreviatus* was inhibited by crude PGIP preparations (Doostdar et al., 1997). Furthermore, in mung bean (*Vigna radiata*), *PGIP* genes were found on a genomic locus that confers resistance to the Phytophaga seed beetles of the genus *Callosobruchus* (Chotechung et al., 2016; Kaewwongwal et al., 2017). Despite these indications, the impact of PGIPs on the interaction between plants and insects remains elusive.

We addressed the impact of PGIPs in an in vivo context by feeding *P. cochleariae* on three *A. thaliana* lines that differed in their PGIP composition. We found a correlation between the PGIP composition and three phenotypic traits of *P. cochleariae* larvae: growth rate, gut PG activity, and *PG* gene expression levels. Growth rate is a fitness parameter, and the performance of *P*. *cochleariae* depends on host plant quality and species (Müller & Müller, 2016, 2017). *Phaedon cochleariae* larvae grew more slowly on wt plants than on both *AtPGIP* knockout mutants, indicating that AtPGIPs lower the food quality of *A*. *thaliana* leaves. Since larval weight gain did not differ between insects fed on either of the *AtPGIP* mutant lines, both AtPGIPs may influence larval development similarly.

Until now, the negative effects of AtPGIPs on eukaryotes have been shown only for fungi and cyst nematodes. Overexpression and loss‐of‐function lines revealed that PGIPs reduce the infection symptoms of the fungi *B*. *cinerea* and *Fusarium* sp. caused by PG inhibition (Ferrari et al., 2003, 2012). The cyst nematode *Heterodera schachtii*, whose survival and reproductive success decrease in the presence of AtPGIP1 (Shah et al., 2017), represents the only example of an in vivo PGIP–fitness correlation in nonfungal eukaryotes. However, the impact of PGIPs on PG activity in vivo and their subsequent fitness consequences in animals are unknown.

We quantified PG activities from larval gut content and showed their increase in larvae fed on *AtPGIP* knockout mutants compared to those fed on wt plants. The absence of AtPGIP1 increased beetle PG activity even more than the lack of AtPGIP2. For the first time, PG activity in the insect gut has been correlated with the food plant's PGIP composition. Given these results and given the impaired larval growth, the significance of the beetle's ability to degrade pectin for its nutrition and development seems clear.

The expression levels of *P. cochleariae GH28*s are consistently influenced by the PGIP composition of the food plant and further confirm the negative impact of AtPGIPs on larval PG activity. GH28‐1 is a highly expressed, active PG enzyme in *P*. *cochleariae,* and its silencing considerably reduces gut PG activity (Kirsch et al., 2014; Kirsch, Kunert, Vogel, & Pauchet, 2019). Although *GH28‐1* was expressed more highly in larvae that fed on wt plants, PG activity was the same or even lower compared with larvae that fed on *AtPGIP* mutants. These results are somewhat counterintuitive, as larvae fed on wt plants possess potentially more PG enzymes due to the elevated *GH28‐1* mRNA level in their guts; however, the larvae showed less PG activity than larvae fed on mutant plants. This discrepancy can be explained by a PGIP‐dependent PG inhibition in such a way that even higher *PG* gene expression cannot compensate for the inhibitory activity of the ingested PGIPs. This hypothesis can only be resolved by showing that *Arabidopsis* PGIPs directly interact with and inhibit beetle PGs, but our attempts in addressing this experimentally failed so far.

*GH28‐1* is not the only *P*. *cochleariae GH28* that is differentially regulated depending on the *A*. *thaliana* line on which the larvae fed. GH28‐2 and GH28‐6 were previously shown to be enzymatically inactive PG pseudoenzymes (Kirsch et al., 2014), and the expression of the corresponding genes is highest in larvae fed on wt plants, though significantly downregulated in larvae that fed on both *PGIP* knockout mutants. Therefore, PGIP composition seems to affect the gene expression level of *GH28s* encoding active PG enzymes as well as inactive PG pseudoenzymes. This work on the PGIP composition of the food plant further supports previous data, indicating that these pseudoenzymes, despite being catalytically inactive, nonetheless contribute somehow to pectin digestion in leaf beetles (Kirsch et al., 2019).

These expression differences suggest that GH28s contribute to compensatory mechanisms induced by a critical shortage of nutrients; such a shortage might be caused by unfamiliar plants or by PGIPs. Similar compensatory responses are known in insects that encounter plant‐derived diets enriched by protease inhibitors, which in turn induce the expression of inhibitor‐sensitive as well as inhibitor‐insensitive proteases (Konarev, 1996; Zhu‐Salzman & Zeng, 2015). Furthermore, in *C. maculatus*, the ingestion of protease inhibitors altered even the gene expression of carbohydrate‐active PCWDEs, including *GH28s* (Chi et al., 2009; Nogueira et al., 2012), illustrating the impact of PCWDEs in insects coping with suboptimal diets.

Plants in turn aim to disarm insects’ adaptations by inducing stress‐responsive defense genes (Agrawal, 1999). Among these are several genes encoding cell‐wall‐protective proteins including PGIPs (Lagaert et al., 2009). The expression of *PGIP* genes is inducible in response to different biotic stressors, such as fungal infection, wounding, and insect feeding (De Lorenzo et al., 2001; Hegedus et al., 2008; Li et al., 2003). The expression of both *AtPGIP* genes was induced after a few hours of caterpillar feeding on rosette leaves but not after aphids heavily infested the plant (Appel et al., 2014; Ehlting et al., 2008). *Phaedon cochleariae* larvae induce the expression of both *AtPGIPs* in wt plants after feeding on their leaves. This confirms that the plant reacts differently to wounding caused by chewing (Lepidoptera and Coleoptera) and piercing (Hemiptera) insects and reveals that *P*. *cochleariae* is exposed to an increasing level of PGIPs while feeding.

Surprisingly, there was a much higher induction of the expression of *AtPGIP1* and *AtPGIP2* genes in the respective mutant plants compared to wt plants in response to beetle feeding which cannot be explained by different steady‐state expression levels. Differences in the induction levels of *PGIP* gene expression in response to beetle feeding can in principle be caused either by genetic compensation (El‐Brolosy & Stainier, 2017) or by differential gene expression between individuals of the same ecotype (Cortijo, Aydin, Ahnert, & Locke, 2018).

Irrespective of the underlying mechanisms, the differences in *PGIP* induction levels between the three lines indicate that the beetles may unexpectedly ingest more PGIP protein when feeding on mutants compared to when feeding on wt plants, as the expression level of the single PGIP in the mutants exceeds that of the sum of both genes in the wt plants. Therefore, our findings suggest that both PGIPs are required to reduce the level of PG activity as well as the extent of larval growth and that such an effect cannot be reached by the high gene expression level of a single PGIP. Support for this hypothesis of synergistic inhibition comes from studies showing, first, that several PGIPs of the same plant inhibit a given PG, and second, that a single PGIP does not inhibit all PGs from a single fungal species (D'Ovidio et al., 2004; Ferrari et al., 2003).

Plants do not always rely on individual, uncoupled defense mechanisms; instead, they may mount defense strategies that act synergistically to reduce the fitness of herbivores. For example, a combination of protease inhibitors and secondary metabolites has been shown to reduce insect performance more efficiently than each component does on its own (Steppuhn & Baldwin, 2007). Glucosinolates (GLS) are secondary metabolites occurring in plants of the family Brassicaceae, the food plants of *P. cochleariae*. The class of glucosinolates comprises more than 120 different compounds and is known to affect insect–plant interactions (Hopkins et al., 2009). Amounts of GLS differ among *A*. *thaliana* ecotypes (Kliebenstein et al., 2001), but whether they also vary among different lines of the same ecotype is unknown. A recent study suggests that GLS induction is impaired in *AtPGIP1*m but not in wt plants in response to infection from nematodes (Shah et al., 2017). Although different GLS profiles in *A. thaliana* food plants did not affect the performance of *P*. *cochleariae* (Mevis & Ulrichs, 2006; Uddin, Ulrichs, Tokuhisa, & Mewis, 2009), to exclude the influence of variations in GLS levels on *P. cochleariae*, we analyzed the GLS profiles of the different *A. thaliana* lines used in the assays. Since no significant differences were found between the lines, we excluded GLS levels as a cause of the observed phenotypical differences.

Not only the efficiency of plant defenses but also the amounts of carbohydrates and proteins available determine the nutritive value of a plant for a herbivorous insect (Le Gall & Behmer, 2014; Schoonhoven et al., 2005). As in the GLS, no significant differences among levels of sugar, amino acids, and essential amino acids were found between the *A. thaliana* lines. Hence, these can also be excluded as causes of variation in larval growth.

In summary, larval performance correlates negatively with PGIP composition and is impaired in the presence of both PGIPs. Moreover, even though the expression of the gene encoding GH28‐1, a highly abundant PG in *P. cochleariae*, is downregulated in larvae feeding on the *AtPGIP* mutants, elevated PG gut activity was observed. Such a seemingly contradictory finding indicates that *A. thaliana* PGIPs may inhibit beetle PG activities, and shows the relevance of pectin degradation for the growth and development of *P. cochleariae*. Still, whether the differences in the weight gain of *P*. *cochleariae* larvae can be explained exclusively by PGIP composition and the corresponding decrease in PG activity or additional effects must be considered, for example, the high energy investment required to elevate the expression of *GH28* genes, remains to be elucidated. Also, efforts to evaluate whether plant PGIPs directly interact and inhibit beetle PGs should be a priority for future studies. In any case, we found in vivo correlative evidence that plant PGIPs may have a negative impact on insects expressing their own PGs. This finding is also intriguing from an evolutionary perspective. The acquisition of a *GH28* gene from a microbial donor by HGT provided the Phytophaga beetles not only with the ability to break down pectin but also with a susceptibility to plant PGIPs. Since *PG* genes have remained in the beetle genomes for about 200 MY, the beneficial effects seem to have outweighed the adverse effects of plant defense. Whether the expansion of *GH28s* into a large gene family is part of the beetle's strategy to cope with plant PGIPs remains under investigation. In the future, we hope to uncover what other counteradaptations herbivorous beetles have developed to protect their pectin‐degrading enzymes.

## CONFLICT OF INTEREST

The authors declare that there are no competing interests.

## AUTHOR CONTRIBUTIONS

R.K. and Y.P. conceived the study and designed the experiments; R.K., E.V., C.S., F.E., T.S., and W.H. performed the experiments; R.K., T.S., and F.E. analyzed the data; and R.K. and Y.P. wrote the paper with contributions from all co‐authors. All authors read and approved the final manuscript.

## Supporting information

Supplementary Material

## Data Availability

The data supporting the results have been archived in the public repository Dryad with the following https://doi.org/10.5061/dryad.8sf7m0cj1
