# Supplementary material for: Plants use identical inhibitors to protect their cell wall pectin against microbes and insects
Source: Ecol Evol. 2020 Mar 12;10(8):3814–24. doi: 10.1002/ece3.6180 (PMC7160172; doi:10.1002/ece3.6180)
Supplement: Supplementary file 1 — Supplementary Material [file ECE3-10-3814-s001.docx]

**Table S1. List of primers used for genotyping of *A*. *thaliana* mutant lines and for RT-qPCR of *A*. *thaliana* *PGIP*s as well as *P*. *cochleariae* *GH28*s.**


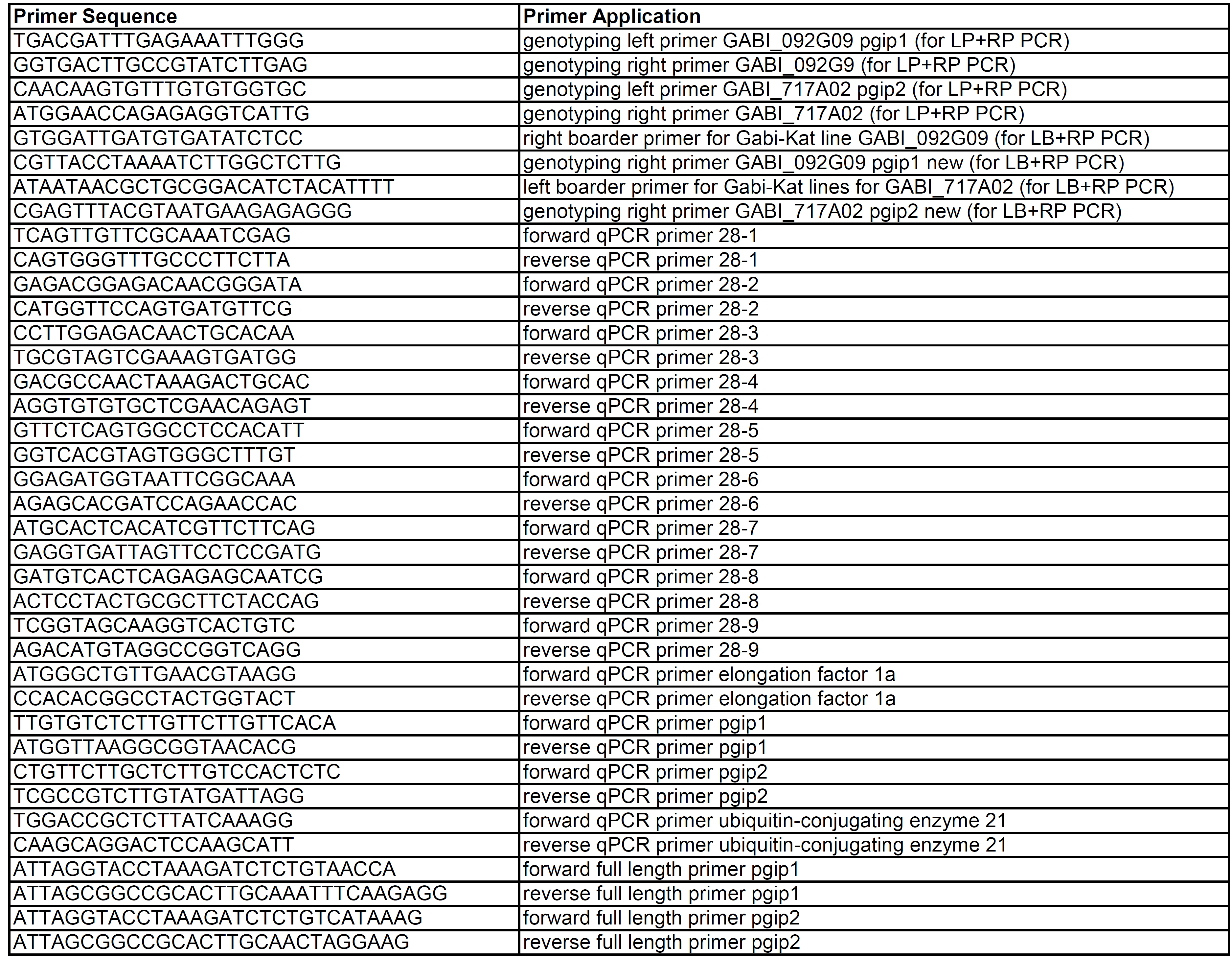


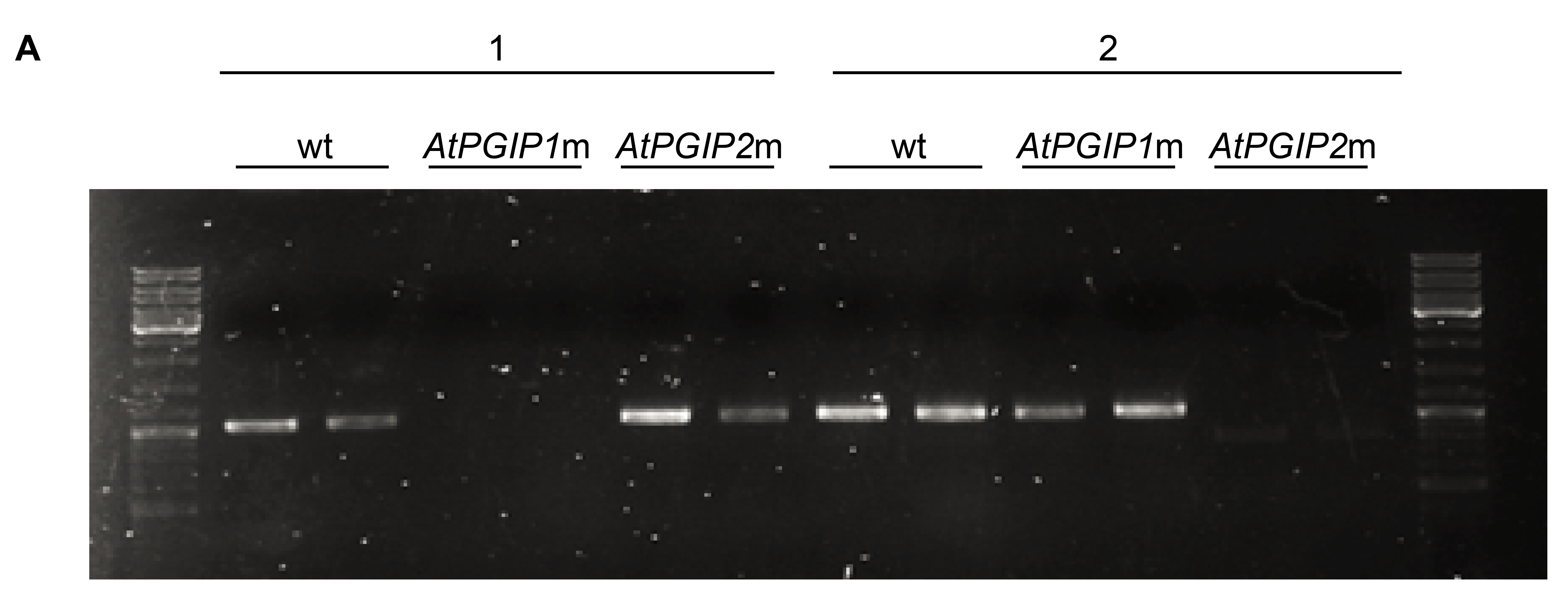


**Figure S1. PCR for presence or absence of *A. thaliana* (A) *pgip* genes and (B) *pgip* transcripts.** Full-length primer targeting (1) *atpgip1* and (2) *atpgip2* were used. The absence of bands indicate the absence of the corresponding (A) wildtype gene and (B) transcript. (A) Gel picture shows an exemplary result taking genomic DNA from two plants per line and *pgip* as a template. (B) Gel picture shows exemplary result taking cDNA pooled from three plants per line and *pgipP* as a template.





**Figure S2. Quantification of GLS in *A. thaliana* wt plants and *PGIP* knockout mutants after *P.* *cochleariae* feeding.** The amount of GLS is expressed as µmol/g fresh weight (FW). No significant differences were detected. Error bars indicate the SEM.





**Figure S3. Quantification of sugars and free amino acids** **in *A. thaliana* wt plants and *PGIP* knockout mutants after *P.* *cochleariae* feeding.** The amount of amino acids is expressed as µmol/g fresh weight (FW) and sugars are given in mg/g FW. No significant differences were detected. Error bars indicate the SEM.
